# Altered composition of the oral microbiome in integrin beta 6-deficient mouse

**DOI:** 10.1080/20002297.2022.2122283

**Published:** 2022-09-12

**Authors:** Osamu Uehara, Jiarui Bi, Deshu Zhuang, Leeni Koivisto, Yoshihiro Abiko, Lari Häkkinen, Hannu Larjava

**Affiliations:** a Faculty of Dentistry, Department of Oral Biological and Medical Sciences, University of British Columbia, Vancouver, BC, Canada; b School of Dentistry, Health Sciences University of Hokkaido, Sapporo, Japan; c Department of Stomatology, The Fourth Affiliated Hospital, Harbin Medical University, Harbin, Heilongjiang, China

**Keywords:** Β6 integrin-null mouse, oral microbiome, 16S ribosomal RNA, QIIME 2

## Abstract

In periodontal disease (PD), bacterial biofilms suppress β6 integrin expression transforming growth factor-β1 signaling, resulting in gingival inflammation and bone loss. β6 integrin-null (*Itgb6^−/−^*) mice develop spontaneous PD. The aim of this study was to unravel potential differences in oral microbiome in wild-type (WT) and *Itgb6^−/−^* FVB mice. Mouse oral microbiome was analyzed from 3- and 6-month-old WT and *Itgb6^−/−^*. The periodontal inflammation and spontaneous bone loss were present in 3-month-old and advanced in 6-month-old *Itgb6^−/−^* mice. The observed amplicon sequence variants (ASVs) of alpha diversity showed close similarity in 3-month-old and 6-month-old *Itgb6^−/−^* mice. Chao1 and ACE methods revealed that the microbiome in *Itgb6^−/−^* mice showed less diversity compared to the WT. UniFrac Principal Coordinate analyses (PCoA) showed a clear spatial segregation and clustering between *Itgb6^−/−^* and WT mice in general, and between 3-month- and 6-month-old WT mice. Weighted PCoA showed the tight clustering and distinct separation of individual mouse samples within *Itgb6^−/−^* and WT. The most abundant microbial classes varied between the sample groups. However, the genus *Aggregatibacter* significantly increased in the 6-month-old *Itgb6^−/−^* mice. β6 integrin-deficient mice develop periodontal inflammation that may relate to dysbiosis in the microbiome that further promotes the disease process.

## Introduction

Complex microbial communities live in the oral cavity, contributing to oral health and diseases [1]. More than 700 different bacterial species have been identified in the human oral cavity [2]. The microbiomes in different niches of the oral cavity, such as saliva, tongue, buccal mucosa, teeth, gingiva and palate, show similarities, although distinct differences are also found [1]. Unique features in the oral microbiome have been reported in many oral diseases, including periodontal disease (PD) [1]. PD is one of the most prevalent oral diseases, resulting in loss of periodontal attachment and destruction of alveolar bone [3]. The colonization of multispecies microbial biofilms on subgingival tooth surfaces is believed to initiate the PD process [4,5]. Certain pathogens, such as *Porphyromonas gingivalis*, can then manipulate the immune system to alter the microbiome towards dysbiosis [6,7]. Mutual interaction of periodontopathogens in the dysbiotic microbiome with the inflammatory response highlights the importance of the host immune system in the disease process [7]. Therefore, genetic and acquired conditions that promote inflammation in the periodontal tissues can also propagate compositional changes in the microbiome.

Integrin αvβ6 is a cell adhesion receptor that is exclusively expressed in epithelial tissues, especially in the barrier epithelia exposed to significant microbial burden, such as the lung, gastrointestinal tract and junctional epithelium (JE) of the gingiva [8]. Integrin αvβ6 binds to the Arg-Gly-Asp (RGD) tripeptide motif in its ligands, including fibronectin, tenascin, vitronectin, some viruses and transforming growth factor-β1 (TGF-β1) and -β3 [8]. It plays an essential role in the innate anti-inflammatory surveillance *in vivo* by activating latent TGF-β1 [8]. Reciprocally, TGF-β1 is required for the constitutive expression of αvβ6 integrin in epithelial cells [9]. Inactivation of the *Itgb6* gene in mice leads to attenuation of the TGF-β1 anti-inflammatory action, resulting in accumulation of inflammatory cells in lungs, skin, gut and periodontal tissues [10–12]. In the gut, αvβ6 integrin-mediated activation of TGF-β1 participates in the retention of dendritic cells, tissue-resident memory T-cells and mast cells [13,14]. This suppression of inflammation plays a role in the prevention of infections by pathogens [13,14]. Interestingly, αvβ6 integrin also positively regulates the AIM2 inflammasome that is crucial for prevention of dysbiosis of the gut microbiome [15,16].

Integrin αvβ6 is strongly expressed in the junctional epithelium between teeth and gingival tissues where it co-localizes with TGF-β1. The importance of αvβ6 integrin in maintaining periodontal health has been demonstrated in the *Itgb6^−/−^* mice that develop spontaneous PD [10,11,15,17]. The progression of PD in *Itgb6^−/−^* mice can be further advanced by polymicrobial infection with human periodontal pathogens [18] or by biofilm accumulation in ligature-induced PD [15,19]. Severe PD in patients with human mutations in the *ITGB6* gene further supports the essential role of this receptor in periodontal health [20]. In PD, JE transforms to pocket epithelium that serves as the last barrier between the subgingival biofilm and the host tissues [21]. During this transition, the expression of αvβ6 integrin is lost [10,22]. *In vitro* experiments demonstrate the role of multi-species oral biofilm in the down-regulation of β6 integrin expression both at mRNA and protein levels via biofilm-induced epidermal growth factor receptor (EGFR)/extracellular signal-regulated kinase (ERK) activation and the subsequent phosphorylation and inhibition of the TGF-β1 signaling mediator Smad3 [9,15,19]. Blocking of EGFR signaling attenuates alveolar bone loss and the inflammatory response in a ligature-induced experimental periodontitis mouse model [19].

Since αvβ6 integrin participates in maintaining the healthy microbiome in the gut, we wanted to explore whether it could similarly affect the oral microbiome, given the fact that its absence is strongly associated with biofilm-induced PD. In the present study, we compared the oral microbiome of wild-type mice to that of *Itgb6-/-* mice using 16S ribosomal bacterial sequencing.

## Materials and methods

### Animals

All animal experiment protocols were reviewed and approved by the University of British Columbia Animal Care Committee (Protocol number A16-0034). Wild-type (WT) and *Itgb6^−/−^* (KO) FVB/NHsd mice were originally obtained from Dr. Dean Sheppard (University of California, San Francisco, CA, USA) [11] and have been raised and maintained for several years in a conventional animal facility at UBC, where they have been tested and determined free of designated pathogens (multiple viruses and certain bacteria), but not necessarily free of all pathogens. The animals were fed Teklad irradiated global soy protein-free extruded diet (Envigo, Indianapolis, IN, USA).

To limit genetic drift, the *Itgb6^−/−^* mice were backcrossed to their WT counterparts every 6–10 generations. The animals used in this study were of the 2nd generation after a backcross. Total of 24 mice were used for the experiment, including six 3-month-old WT mice (4 females and 2 males), six 6-month-old WT mice (4 females and 2 males), six 3-month-old *Itgb6^−/−^* mice (3 females and 3 males) and six 6-month-old *Itgb6^−/−^* mice (4 females and 2 males) (15 females and 9 males in total) (Supplemental Figure S1). The 3-month-old and 6-month-old animals were from subsequent sibling litters born to the same inbred parent pairs. Oral microbes were collected from whole oral swabs of mice as previously described [23]. Maxilla specimens of the mice were then collected and used for the assessment of spontaneous bone loss by micro-computed tomography (μCT) and for inflammation by histochemistry, respectively, as previously described [15,19].

### Micro-computed tomography

The micro-computed tomography was performed as prescribed previously [15]. Briefly, the collected mouse maxillae were fixed in 4% formaldehyde (v/v) in PBS for 24 h at +4°C and then transferred to 2% formaldehyde in PBS for µCT scanning (10 µm voxel size; Scanco CT 40; Scanco, Wayne, PA, USA). Microview Software (Parallax, Ilderton, ON, Canada) was used to analyze the µCT data. The level of alveolar bone loss was evaluated from the horizonal images of µCT. The distances from the cemento-enamel junction to the alveolar bone crest in mesial and distal sites of the second molar in the mouse maxillae were measured.

### Histological assessment of mouse jaw specimens

The histological assessment of jaw specimens was performed as prescribed previously [15]. Briefly, after µCT scanning, the mouse maxilla specimens were decalcified for six weeks in PBS containing 2% formaldehyde and 0.4 M EDTA with two weekly changes of the decalcification solution. The specimens were then rinsed and embedded in paraffin, sectioned (8 µm) in the mesio-distal direction and stained with hematoxylin and eosin. Images were captured under Nikon Eclipse 80i microscope with QIcam camera (Burnaby, BC, Canada) and NIS Elements software (version 4.20; Nikon).

### Oral swabbing and microbial genomic DNA extraction

Individual bacterial oral swabs of six mice per group were obtained from teeth, gingival surfaces, and tongue immediately after sacrifice and stored at −80°C until extraction. Bacterial genomic DNA was extracted using a DNA extraction kit (QIAamp DNA Microbiome Kit, Qiagen, Hilden, Germany) and collected into low-bind tubes (DNA LoBind eppendorf tubes, Eppendorf, Hamburg, Germany). The samples were further purified, concentrated (DNA Clean & Concentrator kit, Zymo Research, Irvine, CA, USA) and stored at −20°C until sequencing.

### V3-V4 amplification and sequencing strategy

The amplicon PCR targeted the V3-V4 regions of bacterial 16S ribosomal RNA (rRNA) gene. Sequencing libraries of the V3-V4 region were generated according to 16S Metagenomic Sequencing Library Preparation instructions (Illumina, San Diego, CA, USA). In brief, V3-V4 regions of 16S bacterial rRNA gene were amplified using a two-step polymerase chain reaction (PCR) protocol (Illumina). The amplicon PCR used KAPA HiFi HS ReadyMix (Kapa Biosystems, Wilmington, MA, USA) and V3-V4 region primers (forward, 5′-TCGTCGGCAGCGTCAGATGTGTATAAGAGACAGCCTACGGGNGGCWGCAG-3′ and reverse, 5′-GTCTCGTGGGCTCGGAGATGTGTATAAGAGACAGGACTACHVGGGTATCTAATCC-3′). The index PCR used KAPA HiFi HS ReadyMix and Nextera XT index kit (Illumina). Libraries were purified using AMPure XP (Beckman Coulter, Indianapolis, IN, USA) and quantified using a Qubit 3 fluorometer (Thermo Fisher Scientific, Waltham, MA, USA). The library was diluted, mixed with PhiX (Illumina) and then subjected to an Illumina MiSeq system for sequencing with a MiSeq reagent kit v3 (600 cycles, Illumina). The sequencing was set up with MiSeq Control Software (Illumina).

### Analysis of sequencing data

Metagenomic sequencing data were analyzed using the software package Quantitative Insights into Microbial Ecology 2 (QIIME2 v2020.2) against the 16S rRNA gene sequences that were assigned to the 16S rDNA database (Greengenes v13.8). Analysis of the amplicon sequence data employed the DADA2 pipeline. To evaluate alpha diversity, Simpson, Chao1, Shannon, Goods coverage, observed features and ACE were used. Statistical significance was set at p < 0.05. The sequencing depth was determined to be 91,349 reads from alpha rarefaction. To evaluate beta diversity, three-dimensional Principal Coordinate Analysis (PCoA) was used to visually compare microbial composition across groups in UniFrac scatterplots. Bacterial community differences with each group were analyzed using the weighted UniFrac and unweighted UniFrac. Statistical significance was set at p < 0.05.

A linear discriminant effect size (LEfSe) analysis for biomarker analysis was performed using the Galaxy web application (http://huttenhower.sph.harvard.edu/galaxy/). Bacterial abundance profiles were calculated at taxonomic levels from phylum to genus in percent abundance using alpha values >0.05 and a logarithmic LDA score > 4.0 as thresholds.

Significant differences in microbial taxon abundance were analyzed using the analysis of composition of microbiomes (ANCOM) in QIIME2. The final significance was expressed as the empirical distribution of W.

### Statistical analysis

One-way ANOVA followed by post hoc comparison with Tukey–Kramer Multiple Comparisons Test was used for the analysis of alveolar bone loss and alpha diversity of 16s RNA gene sequences of the mouse oral microbiome samples (SPSS 24 software; IBM, Chicago, IL, USA). Beta diversity was evaluated based on UniFrac distances representing the fraction of the branch length of the phylogenetic tree that is shared between the groups. Three-dimensional principal coordinate analysis (PCoA) was used to generate UniFrac scatterplots to visually compare microbial compositions across groups. The differences in bacterial communities between the groups were analyzed using the unweighted and weighted UniFrac distance metric. Permutational multivariate analysis of variance (PERMANOVA) was used on the unweighted and weighted UniFrac distance matrix to determine significant differences in microbial communities between the different groups. Statistical significance was set at p < 0.05.

## Results

### *Spontaneous alveolar bone loss and periodontal inflammation in* Itgb6^−/−^ *mice*

**Figure 1.**
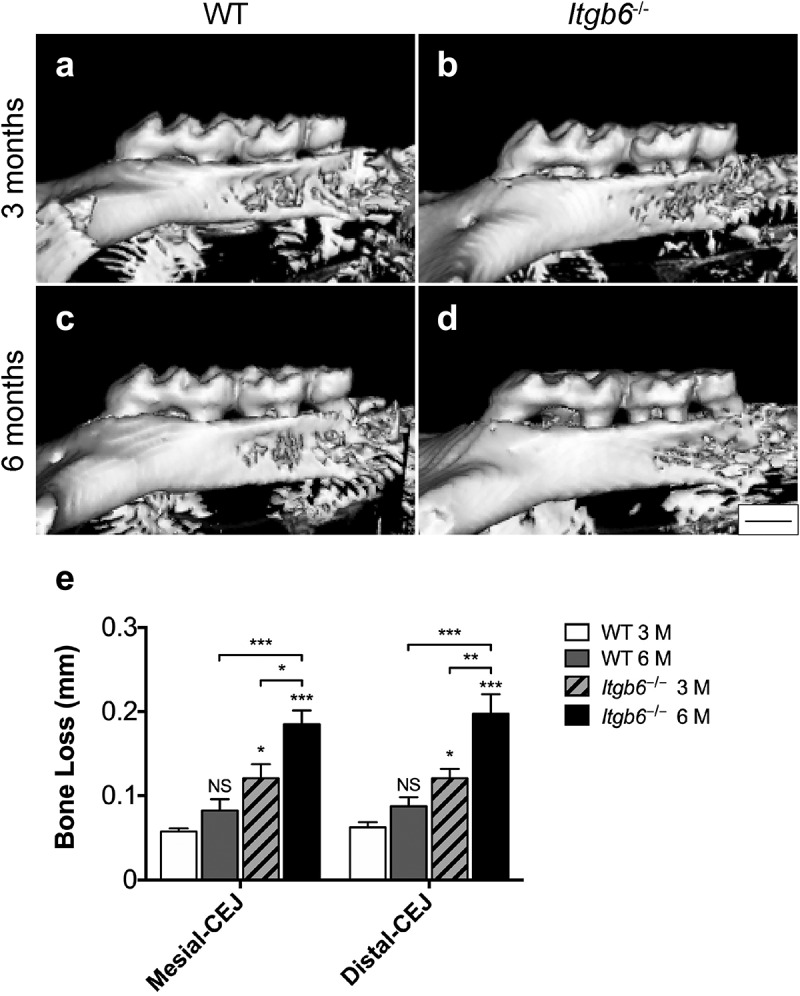
The micro-CT images and analysis of 3- and 6-month-old *Itgb6^−/−^* and WT mouse molar teeth.

**Figure 2.**
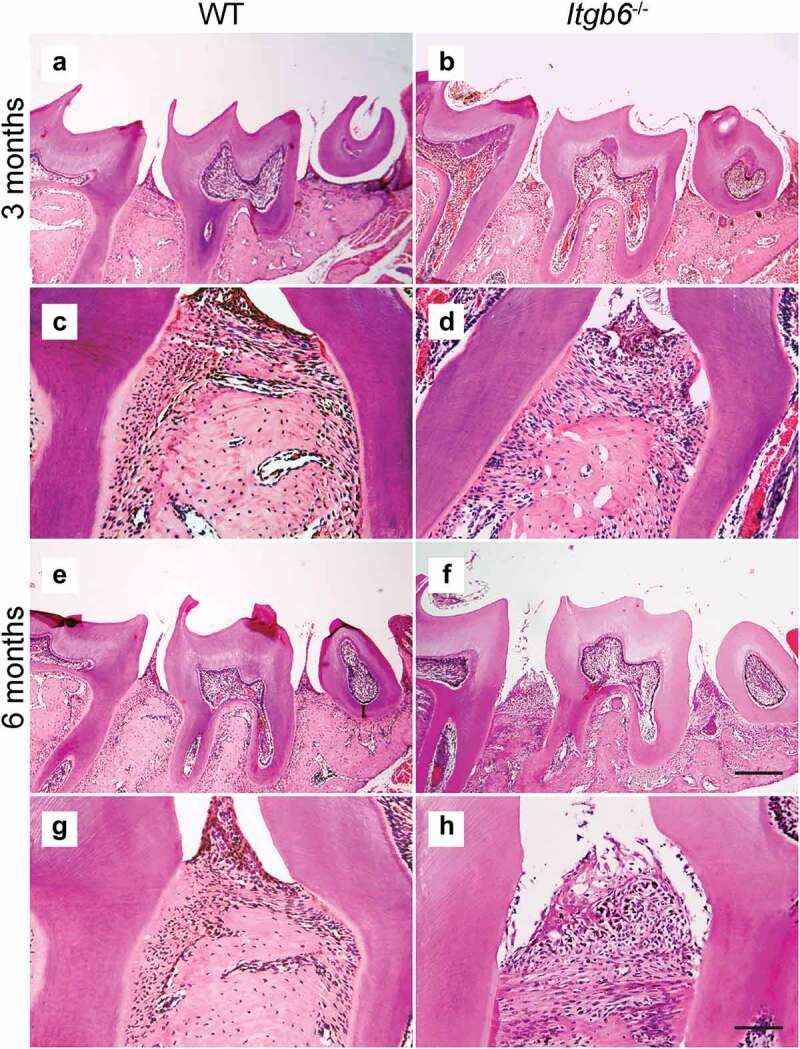
The histological view of periodontal tissues in 3- and 6-month-old *Itgb6^−/−^* and WT mice.

Prior to analysis of oral microbiomes, spontaneous periodontal alveolar bone loss and inflammation were assessed in 3-month-old and 6-month-old WT and *Itgb6^−/−^* mice. The 3-month-old *Itgb6^−/−^* mice showed significant alveolar bone loss in the mesial and distal sites of the maxillary 2nd molars compared to WT mice of the same age (Figure 1a,b and e). Moreover, the bone loss was further advanced in 6-month-old *Itgb6^−/−^* mice, indicating spontaneous progression of bone loss with aging (Figure 1b,d and e). Histological analysis showed normal periodontal architecture in both 3- and 6-month-old WT mice, with the apical end of JE extending to the cemento-enamel junction (CEJ), and minimal inflammation and no bone loss (Figure 2a,c,e and g). In contrast, 3-month and 6-month-old *Itgb6^−/−^*mice displayed disruption of the integrity of JE, inflammatory cell infiltration and loss of alveolar bone (Figure 2b,d,f and h). In addition, JE showed apical extension, especially in the 6-month-old *Itgb6^−/−^* mice (Figure 2f and h). These data indicate that the *Itgb6^−/−^* mice developed spontaneous signs of periodontal disease as they aged.

a-d, Micro-CT scan of the maxillary molar region of 3- and 6-month-old *Itgb6^−/−^* and WT mice. a, 3-month-old WT mouse; b, 3-month-old *Itgb6^−/−^* mouse; c, 6-month-old WT mouse; d, 6-month-old *Itgb6^−/−^* mouse. e, the analysis of vertical distance between the ridge of bone and CEJ in mesial and distal of 2nd molar of 3- and 6-month-old *Itgb6^−/−^* and WT mice. n = 6 animals per group; Mean ± SEM; *, p < 0.05; **, p < 0.01; ***, p < 0.001. Scale bars = 500 μm.

a-h, Representative images of H&E staining of the maxillary 2nd molar region of 3- and 6-month-old *Itgb6^−/−^* and WT mice. a and c, 3-month-old WT mouse; b and d, 3-month-old *Itgb6^−/−^* mouse; eand g, 6-month-old WT mouse; f and h, 6-month-old *Itgb6^−/−^* mouse. a, b, e and f, original magnification ×4; Scale bars = 20 μm; c, d, g and h, show mesial of the maxillary 2nd molar, original magnification ×20; Scale bars = 100 μm.

### *Altered oral microbiome in the* *Itgb6*^−/−^ *mice with PD*

**Figure 3.**
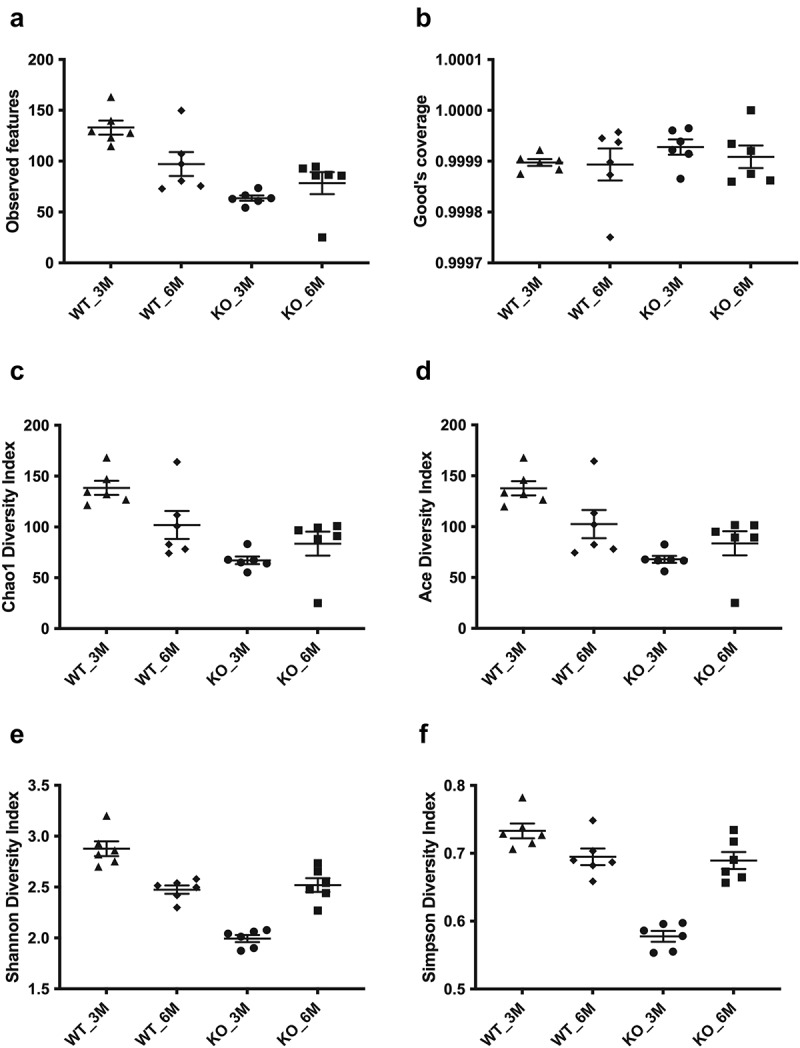
The alpha diversity measurements of the oral microbiome in 3- and 6-month-old *Itgb6^−/−^* and WT mice.

To analyze the potential role of altered oral microbiome for the observed alveolar bone loss and tissue inflammation in *Itgb6^−/−^* mice, oral swabs (teeth, gingival surfaces and tongue) were collected from 3-month and 6-month-old WT and *Itgb6^−/−^* animals and analyzed for their microbial compositions by 16S rRNA gene sequencing. To investigate the differences in oral microbiome diversity between the WT and *Itgb6^−/−^* mice, we used several common alpha diversity indices [24]. In the 3-month-old WT mice, the observed features was 133. This represented the highest observed features in all samples and differed significantly from other sample groups (Figure 3a; WT_3 M vs WT_6 M p < 0.05; vs KO_3 M p < 0.001; vs KO_6 M p < 0.01). In contrast, the observed features was 63.6 and the lowest in the 3-month-old *Itgb6^−/−^* mouse group (Figure 3a). Good’s coverage index showed values above 99% for all mice groups, indicating appropriate sampling (Figure 3b). The Chao1 diversity index was used to estimate the community richness in different samples. In this analysis, the *Itgb6^−/−^* mouse groups ranked lower on the diversity index overall compared to the WT groups (Figure 3c). The 3-month-old WT mouse group showed no statistical difference with the 6-month-old WT mice group in the Chao1 diversity index, however, it showed significant differences compared to the *Itgb6^−/−^* mice groups (Figure 3c; WT3 vs KO3 p < 0.001; vs KO6 p < 0.01). Similar results were obtained when alpha diversity was measured by the ACE diversity index (Figure 3d). Supporting the Chao1 index results, the scores in the *Itgb6^−/-^* mice group showed significant difference as compared to the 3-month-old WT mice group (Figure 3d; WT3 vs KO3 p < 0.001; WT3 vs KO6 p < 0.01). We then used Shannon diversity index to estimate species richness and evenness (more weight in richness) in the oral microbiomes. The Shannon index showed the highest and statistically significant scores in diversity in the 3-month-old WT mice group as compared to the other mice groups (Figure 3e; WT3 vs all the others p < 0.001). Comparably, the 3-month-old *Itgb6^−/−^* mice group showed the lowest score in the Shannon diversity index with statistically significant differences to the other mice groups (Figure 3e; vs all the others p < 0.001). In the 6-month-old groups, however, there was no statistically significant differences between the WT and *Itgb6^−/−^*mouse groups (Figure 3e). Finally, we also used the Simpson’s index that is similar to Shannon index but has more weight on species evenness [24]. In the Simpson’s diversity index analysis, the 3-month-old *Itgb6^−/−^* mouse group showed the lowest and statistically different scores comparing to the other mice groups (Figure 3f; WT3 vs all the others p < 0.001). In summary, the sample diversity scores tended to be the highest in the 3-month-old WT mice group and the lowest in the *Itgb6^−/−^* mouse group, regardless which index was used to estimate species alpha diversity in the samples.

**a**, Scatter dot plot comparing total observed features in the 3- and 6-month-old*Itgb6^−/−^* (KO) mice (KO_3M and KO_6M, respectively) and wide-type (WT) mice (WT_3M and WT_6M); **b**, Good’s coverage; **c**, Chao1 diversity index; **d**, ACE diversity index; **e**, Shannon diversity index; **f**, Simpson diversity index. n = 6 animals per group; Mean ± SEM is presented. Significances for diversity measurements: **A**; WT_3M vs WT_6M p<0.05; vs KO_3M p<0.001; vs KO_6M <0.01; **C**; WT3 vs KO3 p<0.001; vs KO6 <0.01; **D**; WT3 vs KO3 p<0.001; WT3 vs KO6 <0.01; **E**; KO_3M vs all the others p<0.001; **F**; WT3 vs all the others p<0.001.

**Figure 4.**
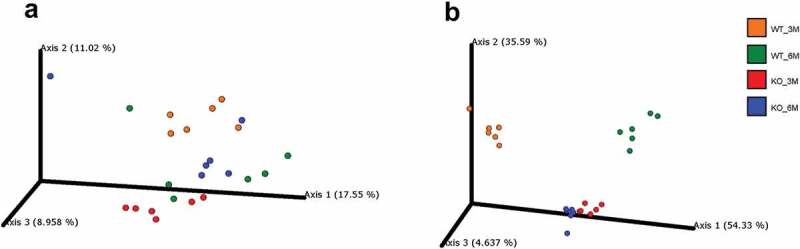
The beta diversity patterns of Principal Coordinate Analysis (PCoA).

To investigate the dissimilarity of the oral microbiomes between the sample groups, we used additional analyses of beta diversity by UniFrac Principal Coordinate analyses (PCoA). In general, a clear spatial segregation and clustering between WT and *Itgb6^−/−^* mice groups were observed. In addition, dissimilarities were also found between the 3-month- and 6-month-old WT mice (Figure 4). Biological replicates from individual mice were highly similar especially in the weighted analysis. The dissimilarities in the unweighted analysis showed significant differences (p < 0.05) between all groups except between 6-month-old *Itgb6^−/−^* and 6-month-old WT, which showed most similarity (Figure 4a). Interestingly, the most dissimilarity in unweighted analysis was between and 3-month-old WT and 3-month-old *Itgb6^−/−^* mice (p = 0.019; Figure 4a). However, these diversities were more pronounced in the weighted (combined abundance and phylogenetic distance) than in the unweighted (phylogenetic distance alone) PCoA analysis (Figure 4). The tight clustering of individual samples within the WT and *Itgb6^−/−^* mouse groups and the distinct separation between *Itgb6^−/−^* and WT oral microbiomes in the weighted PCoA demonstrates that oral microbiomes in the *Itgb6^−/−^* mice are uniquely different from WT mice regardless of age (Figure 4b). Altogether, alpha and beta diversity analyses revealed that oral microbiome in *Itgb6^−/−^* mice with PD was composed of altered microbiota compared to WT mice.

**A**, The PCoA of unweighted UniFrac distances of oral microbiomes in 3- and 6-month-old *Itgb6^−/−^* and WT mice; **B**, The PCoA of weighted UniFrac distances of oral microbiomes in 3- and 6-month-old *Itgb6^−/−^* and WT mice. Each dot represents an individual mouse, and each mouse group is represented by different colors. n = 6 animals per group.

### *Taxonomic composition of the oral microbiome in the* Itgb6^−/−^ *and WT mice*

**Figure 5.**
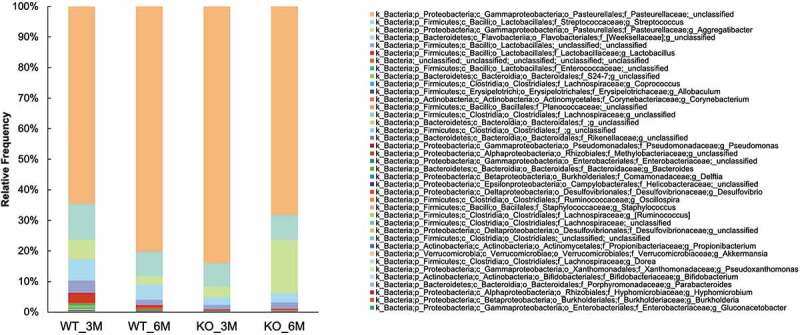
The taxonomic composition of the oral microbiome in 3- and 6-month-old *Itgb6^−/−^* and WT mice.

Next, we used the QIIME 2 database to analyze the genus-level alterations in the bacterial composition between the *Itgb6^−/−^* and WT mice at two different ages. All 24 samples were sequenced using MiSeq, and 7,542,554 total sequences were amplified, ranging from a minimum of 91,349 to a maximum of 409,155 sequences per sample, with a mean of 314,273 sequences per sample. QIIME2 detected a total of 86 different bacterial genera. The oral microbial composition between each individual mouse in different groups was remarkably similar (Figure 5a) with the phylum *Proteobacteria* dominating the samples at 70–90% in all samples (Figure 5). At the family level, *Pasteurellaceae* was the dominating family, followed by *Streptococcaceae* (Figure 5). Members of the *Streptococcaceae* family, considered early colonizers in the dental plaque biofilm [25], showed little differences among the four mouse groups (Figure 5). Supporting the observed differences from the PCoA analyses, the bacterial composition at the genus level showed differences among the four mouse groups (Figure 5). Interestingly, an unidentified bacterial genus from *Rickettsiales* was increased in 6-month-old WT mice but decreased in 6-month-old *Itgb6^−/−^* mice (Figure 5). The *Lactobacillus* from *Lactobacillaceae* family had higher abundance in 3-month-old WT mice compared to others (Figure 5).

The genus level analysis in bacterial composition using the QIIME 2 database between the *Itgb6^−/−^* (KO) and wide-type (WT) mice at ages of 3 and 6 months. **Left**, Taxonomic composition alterations of individual samples of oral microbiome in 3- and 6-month-old*Itgb6^−/−^* (KO_3M and KO_6M) and WT mice (WT_3M and WT_6M); **Right**, The mean of taxonomic composition alterations of oral microbiome in groups of 3- and 6-month-old*Itgb6^−/−^* and WT mice. n = 6 animals per group.

ANCOM was performed in the QIIME2 analysis pipeline to compare the taxonomic abundance of microbiota among the four mice groups. ANCOM analysis showed significant changes in 6 genera (Table 1) among the groups. The high W value (W = 64 to 84) of these bacterial genera suggested that the feature amount had a quantitative difference between the groups. Interestingly, ANCOM showed a significantly higher abundance of 3 bacterial genera in 3-month-old WT mice group as compared to other mice groups.

**Table 1.** The ANCOM results and percentile abundances of features in each group.

The ANCOM results and percentile abundances of features in each group	Median Percentile Abundance				Max Percentile Abundance				W
	WT3M	WT 6M	KO 3M	KO 6M	WT 3M	WT 6M	KO 3M	KO 6M	
k_Bacteria;p_Bacteroidota;c_Bacteroidia;o_Bacteroidales;f_Rikenellaceae;g_Alistipes	73	88	8.5	1	103	299	16	1	84
k_Bacteria;p_Bacteroidota;c_Bacteroidia;o_Bacteroidales;f_Tannerellaceae;g_Parabacteroides	184.5	1	1	187	264	9	1	266	84
k_Bacteria;p_Campilobacterota;c_Campylobacteria;o_Campylobacterales;f_Helicobacteraceae;g_Helicobacter	1	1	1	60.5	1	1	1	84	83
k_Bacteria;p_Desulfobacterota;c_Desulfovibrionia;o_Desulfovibrionales;f_Desulfovibrionaceae;g_uncultured	351.5	34.5	1	26	443	62	1	38	71
k_Bacteria;p_Firmicutes;c_Clostridia;o_Lachnospirales;f_Lachnospiraceae;g_Lachnospiraceae_NK4A136_group	371.5	7.5	1	1	644	62	1	7	68
k_Bacteria;p_Proteobacteria;c_Gammaproteobacteria;o_Enterobacterales;f_Enterobacteriaceae;g_Escherichia-Shigella	98	1	1	1	167	32	1	1	64

**Figure 6.**
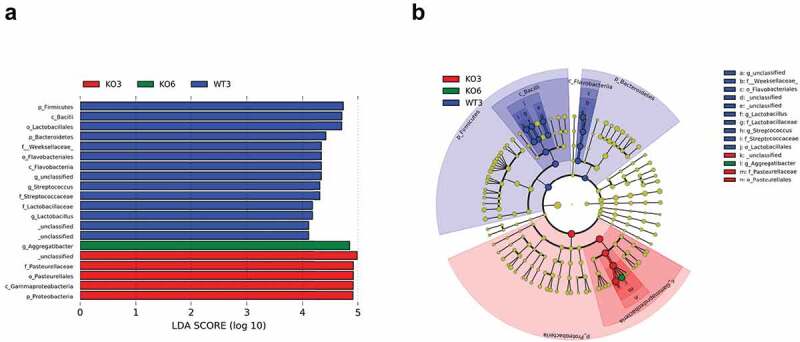
The Linear Discriminant Analysis Effect Size (LEfSe) of 3- and 6-month-old *Itgb6^−/−^*and WT mice.

**Figure 7.**
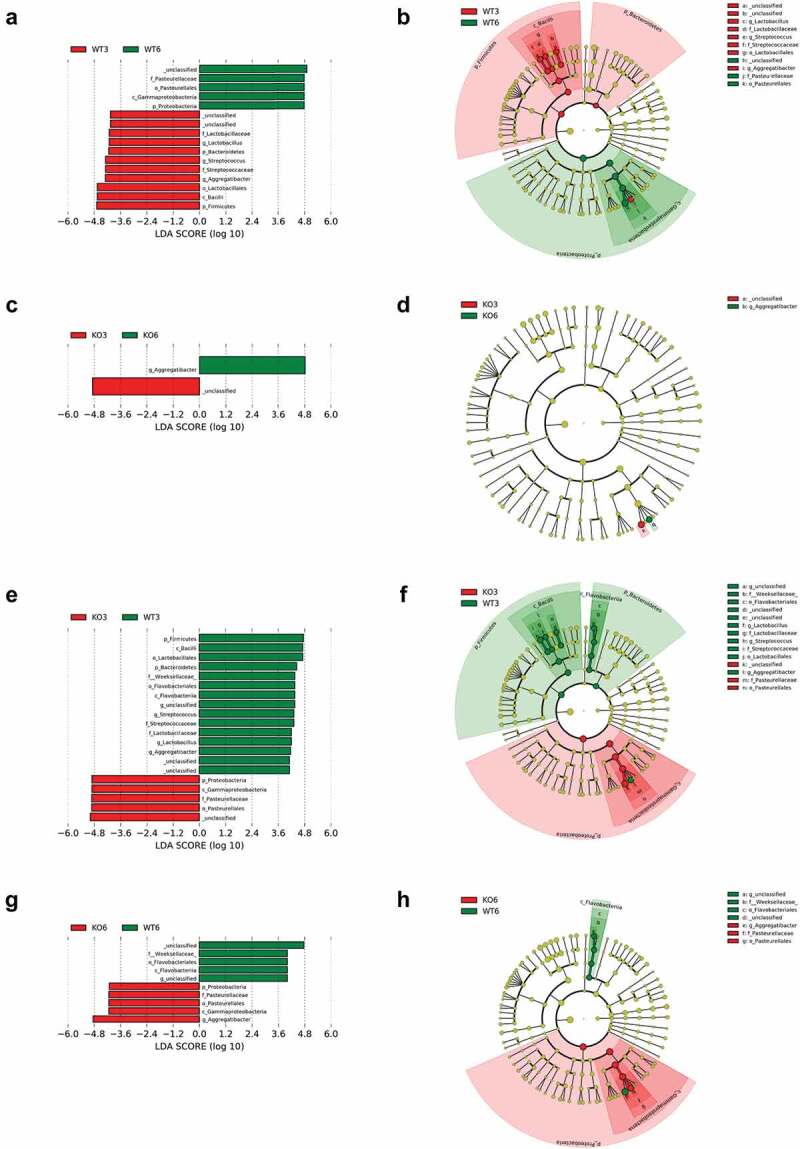
LEfSe analysis of oral microbiomes in 3- and 6-month-old *Itgb6^−/−^* and WT mice.

To identify possible biomarkers associated with each group, we used LEfSe analysis to quantify the magnitude of differences in microbial profiles at taxonomic levels from phylum to genus in percent abundance among the four mouse groups (Figure 6), and between each two mouse groups (Figure 7). In the 3-month-old mouse groups, *Firmicutes* and *Bacteroidetes* phyla were dominant in the WT mice, while *Proteobacteria* phylum dominated in the *Itgb6^−/−^* mice (Figure 7e and f). In the 6-month-old groups, WT mice were abundant with *Cyanobacteria* phylum and *Alphaproteobacteria* class from *Proteobacteria* phylum. The 6-month-old *Itgb6^−/−^* mice were, however, abundant in *Gammaproteobacteria* class from *Proteobacteria* phylum that only includes *Aggregatibacter* genus (Figure 7g and h). In general, WT mice had more abundance in bacterial profiles with a broader range in phyla *Firmicutes, Bacteroidetes, Cyanobacteria* and *Proteobacteria*, including general of *Streptococcus, Lactobacillus, Gluconacetobacter* and three unidentified, compared to the *Itgb6^−/−^* mice, which were only abundant in *Gammaproteobacteria* class under the *Proteobacteria* phylum, including genus of *Aggregatibacter* and unidentified (Figure 6). In paired comparison, in the 3-month-old WT mice, bacteria profiling showed abundance of *Firmicutes* and *Bacteroidetes*, whereas *Proteobacteria* and *Cyanobacteria* were abundant in the 6-month-old WT mice (Figure 7a,b). Interestingly, there were only minor bacterial abundance profile differences at genus level between the 3-month and 6-month-old *Itgb6^−/−^* mice. An unidentified genus was abundant in the 3-month-old *Itgb6^−/−^* mice and *Aggregatibacter* was abundant in 6-month-old *Itgb6^−/−^* mice (Figure 7c,d). These genera were both from *Pasteurellaceae* family. However, opposite findings were observed in WT mice, in which *Aggregatibacter* was abundant in the 3-month-old group, while unidentified genus was abundant in the 6-month-old group (Figure 7a,b). In the genus *Aggregatibacter*, the only member that was identified was *A. pneumotropica*.

LEfSe analysis was used to compare the differences of bacterial profiles at taxonomic levels from phylum to genus in percent abundance among 3- and 6-month-old*Itgb6^−/−^* (KO3 and KO6) and WT mice (WT3 and WT6). **a**, The bar graph of LEfSe analysis among 3- and 6-month-old*Itgb6^−/−^* and WT mice. Linear discriminant analysis (LDA) scores > 4. n = 6 animals per group; **b**, The cladogram of LEfSe analysis among 3- and 6-month-old*Itgb6^−/−^* and WT mice. Linear discriminant analysis (LDA) scores > 4. n = 6 animals per group.

LEfSe analysis was used to compare the differences of bacterial profiles at taxonomic levels from phylum to genus in percent abundance between 3- and 6-month-old*Itgb6^−/−^* (KO3 and KO6) and WT mice (WT3 and WT6). a and b, LEfSe analysis between 3- and 6-month-old WT mice; c and d, LEfSe analysis between 3- and 6-month-old*Itgb6^−/−^* mice; e and f, LEfSe analysis between 3-month-old*Itgb6^−/−^* and WT mice; g and h, LEfSe analysis between 6-month-old*Itgb6^−/−^* and WT mice. All linear discriminant analysis (LDA) scores > 4. n = 6 animals per group.

## Discussion

The present study demonstrates that oral microbiome is altered in mice that lack the JE-specific αvβ6 integrin known to regulate anti-inflammatory TGF-β1 activity [8]. Differences are already seen in 3-month-old mice, and the microbiome continued to be different in 6-month-old mice with progressive periodontal inflammation and bone loss in the *Itgb6^−/−^* mice. To our knowledge, this report is the first one to show that the reduced anti-inflammatory action in the JE is associated with microbial shifts associated with PD.

Murine oral microbiome has been recently curated [26]. In general, laboratory mouse oral microbiome seems to show high variations between different strains and also lower diversity than that of wild mice [26]. In laboratory mice, oral microbiomes appear to vary in microbial composition depending on tooth eruption, age and mouse strain vendors [27]. In a study comparing oral microbiomes in the same strain (C57BL/6) obtained from different vendors, mice from The Jackson Laboratory (Jax mice) and from the Taconic Biosciences (Tac mice) significantly differed in their oral microbiome composition. The Tac mice harbored *Lactobacillus* species as the dominant species with little changes with aging while the oral microbiome in the Jax mice was more diverse and shifted over time [27]. Co-housing the mice caused horizontal transfer of oral microbiomes towards Tac microbiome. To our knowledge, the oral microbiome of the FVB/NHsd mice used in the present study has not been previously reported. One limitation of the present study is that the *Itgb6^−/−^* mice were bred separately after they were backcrossed to the WT, albeit being housed in the same facility in the same room and fed an identical diet. However, co-housing the WT and *Itgb6^−/−^* mice could have potentially resulted in horizontal transfer of the microbiomes, as seen in the previous study and, therefore, confused the results. Unlike in the Jax and Tac mice of the previous study, the normal flora of the FVB/NHsd mice was dominated by *Pasteurellaceae* and *Streptococcaceae*. Although the dominant species of oral microbiome in the WT FVB/NHsd mice did not change from 3 to 6 months, there were significant changes in the diversity and composition, suggesting that shifts in the less dominant microbial genera are common in healthy oral microbiome. One limitation of our study that needs to be considered when comparing 3- and 6-month-old mice was cross-sectional sampling of the microbiomes that may not perfectly represent longitudinal changes in individual mice. However, age-related changes in oral microbiome in C57BL/6 mice have been reported in another study with *Streptococcus* dominating the younger (2-month-old) mice while *Neisseria* dominating the older animals (15-month-old) with high inter-individual differences [28]. Overall, oral microbiomes related to health (commensal bacteria) seem to be highly variable. Interestingly, even commensal oral microbiota could be associated with regulation of alveolar bone homeostasis [28].

The *Itgb6^−/−^* mice groups with periodontitis tended to have the lowest diversity, especially within the 3-month-old mice group. Reduced diversity together with a loss of beneficial and an expansion of pathogenic microbes have been classified as key factors leading to dysbiosis [29]. However, studies have reported either higher, lower or unchanged diversities in PD compared to health [reviewed in 6]. The differences could relate to the sampling and disease state of the periodontal pockets in PD. Interestingly, the alpha diversity in gingivitis appears to be higher than in PD, suggesting reduction in diversity when gingivitis transforms to PD [6]. All human studies have analyzed the subgingival microbiomes while our data was composed of the total oral microbiome, and they cannot, therefore, be directly compared. Subgingival collection of microbiological specimens from mice is not feasible due to the size of the dentition. Although site-specific microbiomes exist in different parts of oral cavity and saliva, they also share similarities [30]. Periodontal pathogens are not limited to the subgingival environment but are also found on mucous membranes. For example, the presence of *P. gingivalis* and *Aggregatibacter actinomycetemcomitans* on tongue and saliva microbiomes correlates with a higher risk of having PD [31,32]. Other studies have also demonstrated that tongue and buccal mucosal microbiomes are different in patients with PD compared to healthy controls [33,34]. Based on these human studies, the oral microbiome samples of the present study composed of swabs of teeth, gingiva and tongue should sufficiently represent the oral microbiome related to periodontal health and disease.

The biggest difference in abundance between the 6-month-old WT and the *Itgb6^−/−^* mice was in Genus *Aggregatibacter* that showed strikingly higher abundance in the *Itgb6^−/−^* mice. *Aggregatibacter* genus contains a well-known human periodontal pathogen *A. actinomycetemcomitans* [35]. The only member of the *Aggregatibacter* genus identified in our analyses was *A. pneumotropica*. Literature about this species is limited to a few papers where it has been found in the gut microbiome [36–38]. Interestingly, in the intestinal epithelium in specific autophagy 5-deficient mice (*Atg5^−/−^*), the relative abundance of *A. pneumotropica* was significantly increased in the jejunum-ileum part of the gut [38]. *Atg5* serves as an important regulator of intestinal inflammation and antibacterial defense [39,40]. Like intestinal epithelial cells, JE cells in the gingiva are constantly exposed to diverse commensal microbiome and occasionally to pathogenic bacteria. Altered regulation of inflammation in both tissues could thus favor the outgrowth of the pathogens and dysbiosis of the biofilm. Accordingly, αvβ6 integrin-mediated activation of TGF-β1 in the gut participates in retention of dendritic cells, tissue-resident memory T-cells and mast cells [13,14]. Intestinal epithelium-derived TGF-β1 is considered as the master regulator of gut inflammation and microbiome [41] and could, therefore, play a similar role in the JE, although reduced activation of TGF-β1 in JE has not been directly demonstrated. Interestingly, gingival tissue in the *Itgb6^−/−^* mice has reduced expression levels of AIM2 and interleukin-18 that appear to be directly regulated by the αvβ6 integrin – TGF-β1 pathway [15]. AIM2 inflammasome plays a crucial role in the homeostasis of normal gut microbiome via IL-18 activation [42]. The present study suggests that periodontal inflammation caused by αvβ6 integrin-deficiency can lead to significant alterations in the oral microbiome that could be related to dysregulation of inflammasomes. Many microbial species associated with periodontitis are also found in health [6], supporting the notion that they become enriched in inflammatory conditions.

Findings of the present and previous studies corroborate the concept that cross-talk between host inflammatory signals and microbiome plays an important role in the pathogenesis of PD.

## Supplementary Material

Supplemental Material
