# Supplementary figures and images for: Altered composition of the oral microbiome in integrin beta 6-deficient mouse
Source: J Oral Microbiol. 2022 Sep 12;14(1):2122283. doi: 10.1080/20002297.2022.2122283 (PMC9481083; doi:10.1080/20002297.2022.2122283)

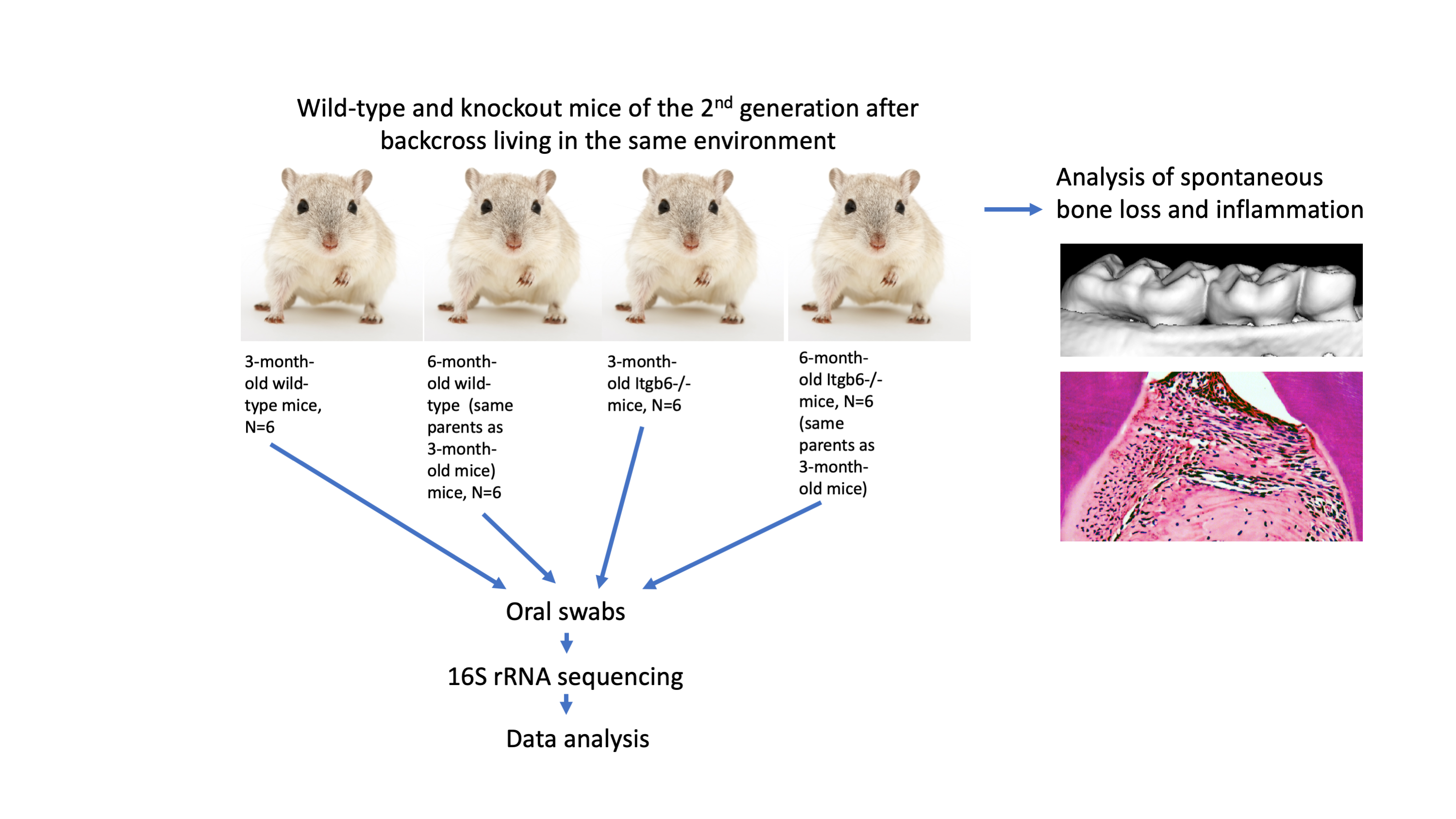

Supplement: Supplemental Material [file ZJOM_A_2122283_SM6777.tiff]
